# Pattern of oral anticoagulant prescribing for atrial fibrillation in general practice: an observational study in The Netherlands

**DOI:** 10.3399/BJGPO.2022.0179

**Published:** 2023-02-22

**Authors:** Catharina CM Kager, Maaike Horsselenberg, Joke C Korevaar, Cordula Wagner, Karin Hek

**Affiliations:** 1 Netherlands Institute for Health Services Research (NIVEL), Utrecht, The Netherlands; 2 Amsterdam Public Health Research Institute, Amsterdam UMC, Location VUmc, Amsterdam, The Netherlands

**Keywords:** anticoagulants, atrial fibrillation, prescribing pattern, general practice, direct-acting oral anticoagulant, vitamin K antagonist, primary healthcare

## Abstract

**Background:**

In the Dutch atrial fibrillation (AF) guideline for GPs, vitamin K antagonists (VKAs) and direct oral anticoagulants (DOACs) are seen as equivalent, while in cardiology there is a preference for DOACs.

**Aim:**

To describe the pattern of oral anticoagulant (OAC) prescribing for AF by GPs and assess whether GPs proactively convert between VKAs and DOACs in patients with AF.

**Design & setting:**

Observational study using routine practice data from 214 general practices, from 2017 until 2019.

**Method:**

Patients aged ≥60 years diagnosed with AF, who had been prescribed OACs by their GPs in 2018 were included. A distinction was made between starters, who were participants who did not use OACs in 2017, and prevalent users. It was observed and recorded whether patients switched between VKAs and DOACs.

**Results:**

A total of 12 516 patients with AF were included. Four hundred and seventy-six patients (4%) started OACs in 2018; 12 040 patients were prevalent OAC users. When GPs started patients on OACs, DOACs were prescribed the most (88%). Among prevalent users, more than half of the patients used VKAs (60%). GPs switched between OACs for 1% of starters and 0.6% of prevalent users in 2018 and 2019.

**Conclusion:**

Dutch GPs predominantly start with DOACs in newly diagnosed patients with AF. Prevalent patients predominantly use VKAs and switching from a DOAC to a VKA is unusual. Consequently, the number of patients using VKAs will decline in the upcoming years. This trend raises questions about the future of organising frequent international normalised ratio (INR) checks for VKA users.

## How this fits in

In the Dutch guideline for GPs, VKAs and DOACs are seen as equivalent, unlike the guideline for cardiologists where DOACs are advised. It is not known yet if Dutch GPs have preference for either VKAs or DOACs when prescribing OACs for patients with AF. This study fills that knowledge gap, and provides important information that needs to be acted on when organising future check-ups of OACs for patients with AF.

## Introduction

AF is the most common heart rhythm disorder worldwide^1^ and is associated with a high mortality and morbidity risk.^2^ It is estimated that in 2060 there will be 14.4 million patients in the EU with AF^3^ and that 3.2% of the Dutch population will have AF.^4^ To compare, in 2019 2.6% of the total Dutch population had AF.^5^

The vast majority of all patients with AF use medication to prevent thromboembolism (TE). VKAs have long been the drug of choice in the long-term treatment and prevention of TE in patients with AF.^6,7^ Since the introduction of DOACs in 2008, there has been a wider choice of prescribing OACs. VKAs have a small therapeutic window, and therefore frequent INR checks are necessary to adjust the dosage. These checks can be provided in primary or secondary care or by specialised thrombosis services. In The Netherlands, monitoring of patients using VKAs is done by specialised thrombosis services. For DOAC users there is not a national monitor for check-up in The Netherlands. DOACs are provided in a fixed dose and therefore regular INR checks are no longer necessary, but factors such as renal function, weight, and age can make a dosage adjustment necessary^8^ and at least a yearly check-up is advised to prevent under or overdosing. DOACs come with a more favourable side-effect profile. Compared with VKA users, DOAC users had a significant reduction in stroke, intracranial haemorrhage, and mortality, and with similar number of major bleeding as for the VKA warfarin. However, DOACs increased the risk of gastrointestinal bleeding compared with VKAs.^9,10^ Therefore, DOACs are contraindicated for patients with a medical history of gastrointestinal bleeding. Also, frail older people, patients who are at risk of developing acute renal failure^11^ and patients with mechanical heart valves^12^ should not use DOACs.

The European guideline for cardiologists states that if a patient with an indication for OACs is eligible for a DOAC, this is preferable to a VKA.^1^ The UK guideline (2021) for primary care on AF advises to offer a VKA only when DOACs are contraindicated.^13^ In Denmark, European guidelines for cardiology on this matter are followed, with the modification that VKA is also recommended if the time in the therapeutic range (when using a VKA) is 70% or more.^14,15^ In the Dutch guidelines for GPs from 2017, VKAs and DOACs are seen as equivalent when starting OACs for AF if there are no contraindications.^16,17^ Since in The Netherlands GPs function as gatekeepers to healthcare services and many GPs have access to an electrocardiogram (ECG; needed for diagnosing AF), they are able to diagnose AF.^18^

Since the introduction of DOACs in the EU in 2008, large variations of DOAC uptake were observed between European countries.^19^ A cross-national drug utilisation study on AF among six Western European countries (2008–2015) showed an increased incidence of use of DOACs related to AF in the study period across Denmark, Germany, Spain, UK, France, and The Netherlands. In The Netherlands a slower DOAC uptake than in most other Western European countries was found.^20^ This might be because of a report, published in 2012, from the Health Council of The Netherlands. The report advised a careful introduction of DOACs, given the lack of real-world data, absence of specific antidotes, and a substantial risk of non-compliance owing to a lack of monitoring.^21^ Nowadays, for three out of four DOACs an antidote is available.

The Dutch guideline regarding treatment for AF and the availability of new antidotes might influence the first choice of OAC treatment for AF provided by GPs. This study describes which type of OAC is prescribed by GPs for patients newly diagnosed with AF and for patients already treated for AF. It will look at whether there are differences between patients using the DOACs or VKAs. Moreover, it will study whether GPs switch from one type of OAC to the other. More knowledge about prescribing behaviour regarding OACs in primary care can help GPs to anticipate necessary check-ups for patients using OACs and the organisation of these check-ups.

## Method

### Study population

The data were derived from the Nivel Primary Care Database (Nivel-PCD), which consists of data from routine electronic health records of a large pool of general practices in The Netherlands.^22^

The participating practices are spread throughout the country and the population covered is representative on age and sex to the national Dutch population. The database includes information on patient sex, year of birth, dates of consultation, and clinical diagnoses, which are coded using the International Classification of Primary Care version 1 (ICPC-1) scheme.^23^ In addition, information on prescriptions by physicians is available, which is coded according to the Anatomical Therapeutic Chemical (ATC) classification index.

Practices were included in the study from 2017–2018, with follow-up in 2019, if at least 70% of consultations included a registered diagnosis, and prescription and morbidity data were registered for at least 46 weeks of the year. Practices that did not comply with this were excluded from the dataset. The study population consisted of all patients aged ≥60 years who were diagnosed with AF and had a prescription of a DOAC or VKA in 2018 and were registered in their GP practice in the complete study timeframe (January 2017–December 2018). Covariates are mentioned in Supplementary Table S1. GPs electronic health records may also include prescriptions from specialists. The study aimed to assess only prescriptions by GPs. Therefore, prescriptions were only included with a contact with the GP practice on the same day of the prescription or a contact on the day before the prescription (this could also be a telephone contact). A sensitivity analysis was conducted in a subset of practices in which the prescriber for the researchers was known to verify if this gave similar results.

Patients who did not have any prescription for OAC in 2017 and (like all patients of the study) had a prescription of an OAC in 2018 were defined as ‘starters’. For assessing switching of starters, patients were selected who could be followed for a year after their first OAC prescription. Therefore, 2019 was used as a follow-up year. If a patient who started a VKA switched to a DOAC, the research team observed whether these patients received a prescription for a DOAC after their initial VKA prescription from 2018 in the 12 months following the initial VKA prescription. For assessing if a DOAC starter switched to a VKA, the research team recorded whether, after their initial DOAC prescription in 2018, they received a VKA prescription in the 12 months following the initial DOAC prescription. Prevalent users were defined as patients who already had a prescription for either a DOAC or VKA in 2017. For assessing if these prevalent patients switched OAC, it was noted whether these patients switched to another OAC after their first prescription in 2018. For a successful switch, the new medication had to be used for at least 3 months.

### Patient characteristics

Age, sex, and renal function (estimated glomerular filtration rate [eGFR] (ml/min/1.73 m^2^) were assessed, next to other comorbidities and comedication (detailed in the Supplementary file).

### Statistical analyses

Stata (version 16.1) was used to analyse the data using descriptive methods. Differences in the characteristics were assessed between VKA and DOAC starters, between prevalent VKA users and prevalent DOAC users, and between prevalent VKA users who did and who did not switch to a DOAC using Pearson’s χ^2^ for categorical variables and independent samples *t*-tests for continuous variables. A *P* value <0.05 was considered significant.

## Results

Overall, 14 068 patients had AF and used OAC in 2018. Of these 12 040 were prevalent OAC users and 2028 were starters, of whom 1552 were excluded as they did not meet the criterion of starting OAC by the GP (characteristics provided in Supplementary Table S2). Therefore, a total of 12 516 patients were included from 214 general practices. (Table 1)

**Table 1.** Characteristics of patients with atrial fibrillation (prevalent oral anticoagulant [OAC] users and patients who started an OAC by the GP)

Patient characteristics	*n* = **12 516**
Age (years, mean, sd)	77.2±8.0
Sex (% female)	45.4
Type of OAC (% DOAC)	43.1
Type of user (% starter)	3.8
**Comorbidity**	
Thrombosis (%)	1.5
Embolism (%)	0.8
CVA or TIA (%)	14.3
Heart failure (%)	18.3
Depression (%)	3.4
**Renal function**	
eGFR ≥50 (%)	66.6
eGFR <50 en≥30 (%)	14.2
eGFR <30 (%)	2.6
eGFR n.a. (%)	16.6
**Comedication**	
Antidiabetic drugs (%)	20.7
Cardiovascular drugs (%)	95.5
Antidepressants (%)	13.3
Benzodiazepines (%)	17.0
Gastrointestinal drugs (%)	65.1
No comedication (%)	1.8

CVA = cerebrovascular accident. DOAC = direct oral anticoagulant. eGFR = estimated glomerular filtration rate. OAC = oral anticoagulant. TIA = transient ischaemic attack.

### Anticoagulant treatment to starters

A total of 476 patients of 14 068 patients with AF were started with OACs by their GPs in 2018, their characteristics are described in Table 2. Most patients who started OACs in general practice by their GPs started with DOACs (88%). VKA starters were older, had a higher percentage of heart failure comorbidity, and used more antidepressants and gastrointestinal drugs compared with patients starting DOACs.

**Table 2.** Characteristics of patients who started a VKA versus a DOAC in 2018 with a first OAC prescription by a GP

Starters OAC in 2018	VKA		DOAC		*P* value
*n*	57 (12%)		419 (88%)		
Mean age, years, SD	79.4±8.9		75.1±7.9		<0.01
	*n*	%	*n*	%	
Sex					
Male	24	42.1%	207	49.4%	0.30
Female	33	57.9%	212	50.6%	0.30
Type of DOAC					
Apixaban	–		123	29.4%	
Dabigatran	–		113	27.0%	
Edoxaban	–		36	8.6%	
Rivaroxaban	–		147	35.1%	
Renal function (ml/min/1.73 m^2^)				–	<0.01
eGFR ≥50	32	56.1%	333	79.5%	
eGFR <50 and ≥30	15	26.3%	37	8.8%	
eGFR <30	5	8.8%	0	–	
eGFR n.a.	5	8.8%	49	11.7%	
Comorbidity					
Previous thrombosis	0	–	7	1.7%	0.33
Previous embolism	0	–	0	–	–
Prior stroke or TIA	7	12.3%	30	7.2%	0.18
Heart failure	7	12.3%	19	4.5%	0.02
Depression	3	5.3%	12	2.9%	0.33
Comedication					
No	1	1.8%	15	3.6%	0.47
Antidiabetic drugs	11	19.3%	61	14.6%	0.35
Cardiovascular drugs	51	89.5%	390	93.1%	0.33
Antidepressants	12	21.1%	44	10.5%	0.02
Benzodiazepines	13	22.8%	57	13.6%	0.07
Gastrointestinal drugs	43	75.4%	252	60.1%	0.03

DOAC = direct oral anticoagulant. eGFR = estimated glomerular filtration rate. TIA = transient ischaemic attack. VKA = vitamin K antagonist.

A sensitivity analysis in a subset of 104 practices, in which the prescriber was known, showed that the GP started DOACs in 83% and VKAs in 17% of patients. This analysis matched the study's findings using all 214 practices, in which it was assumed OACs were prescribed by GPs if a consultation with the GP was scheduled on the same day or the day before the prescription (Table 2: 88% started DOAC, 12% started VKA).

**Figure 1.**
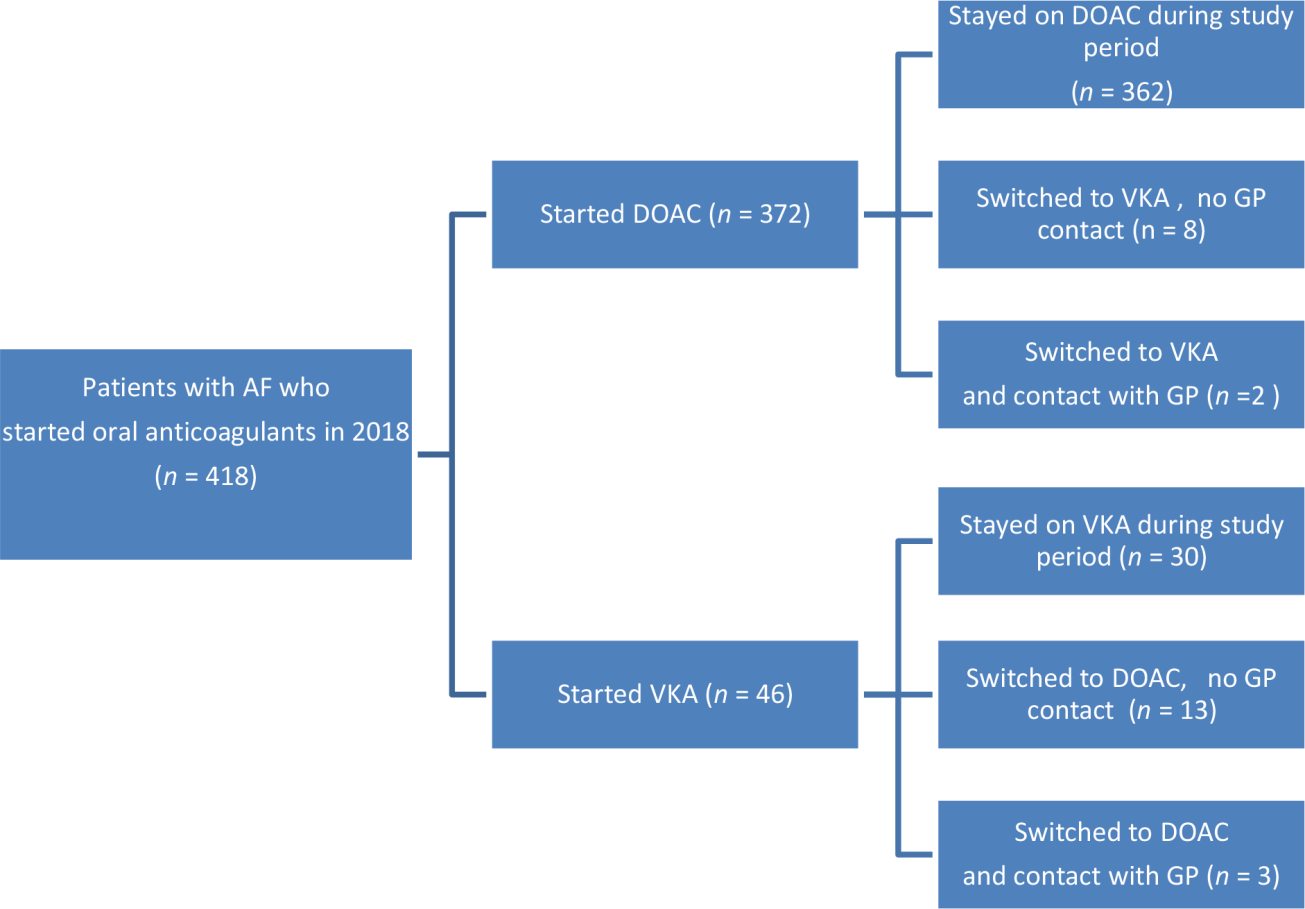
Patients who were newly diagnosed with atrial fibrillation (AF) in 2018 and started a vitamin K antagonist (VKA) or direct oral anticoagulant (DOAC); prescribed by their GP and followed for 1 year to assess switching rate

Of all patients who started an OAC in 2018 and who could be followed for 1 year after their initial prescription (418 patients), only a small group of patients switched to another anticoagulant (6%; *n* = 26/418). For only five patients was this switch likely to have been initiated by their GP (1%; *n* = 5/418) (Figure 1).

### Anticoagulant treatment to prevalent users

A total of 12 040 patients already used OAC in 2018 (prevalent users). Their characteristics are described in Table 3. Sixty per cent of prevalent users used VKAs (*n* = 7183/12 040). Prevalent VKA users were older, had a higher percentage of heart failure comorbidity, and used more antidepressants and gastrointestinal drugs compared with prevalent users of DOACs.

**Table 3.** Characteristics of patients who already used OAC in 2018; prevalent vitamin K anticoagulant (VKA) users versus prevalent direct oral anticoagulant (DOAC) users

Characteristics	**Prevalent OAC users**				
	Prevalent VKA users		Prevalent DOAC users		*P* value
*n*	7183		4857		
Mean age, years, SD	78.6±8.0		75.3±7.6		<0.01
			
	*n*	%	*n*	%	
Sex					0.01
Male	3868	53.8%	2732	56.2%	
Female	3315	46.2%	2125	43.8%	0.01
Type of DOAC^a^					
Apixaban	–		1281	26.4%	
Dabigatran	–		1464	30.1%	
Edoxaban	–		286	5.9%	
Rivaroxaban	–		1821	37.5%	
Renal function (ml/min/1.73 m^2^)					<0.01
eGFR ≥50	4605	64.1%	3366	69.3%	
eGFR <50 and≥30	1190	16.6%	529	10.9%	
eGFR <30	259	3.6%	56	1.2%	
eGFR n.a.	1129	15.7%	906	18.6%	
Comorbidity					
Previous thrombosis	117	1.6%	65	1.3%	0.20
Previous embolism	67	0.9%	34	0.7%	0.17
Prior stroke or TIA	1097	15.3%	654	13.5%	0.01
Heart failure	1661	23.1%	605	12.5%	<0.01
Depression	252	3.5%	160	3.3%	0.53
Comedication					
No	117	1.6%	97	2.0%	0.13
Antidiabetic drugs	1651	23.0%	869	17.9%	<0.01
Cardiovascular drugs	6902	96.1%	4611	94.9%	<0.01
Antidepressants	983	13.7%	619	12.7%	0.14
Benzodiazepines	1307	18.2%	749	15.4%	<0.01
Gastrointestinal drugs	4621	64.3%	3234	66.6%	0.01

^a^Type of DOAC unknown for five patients. DOAC = direct oral anticoagulant; eGFR = estimated glomerular filtration rate. OAC = oral anticoagulant. TIA = transient ischaemic attack. VKA = vitamin K antagonist.

**Figure 2.**
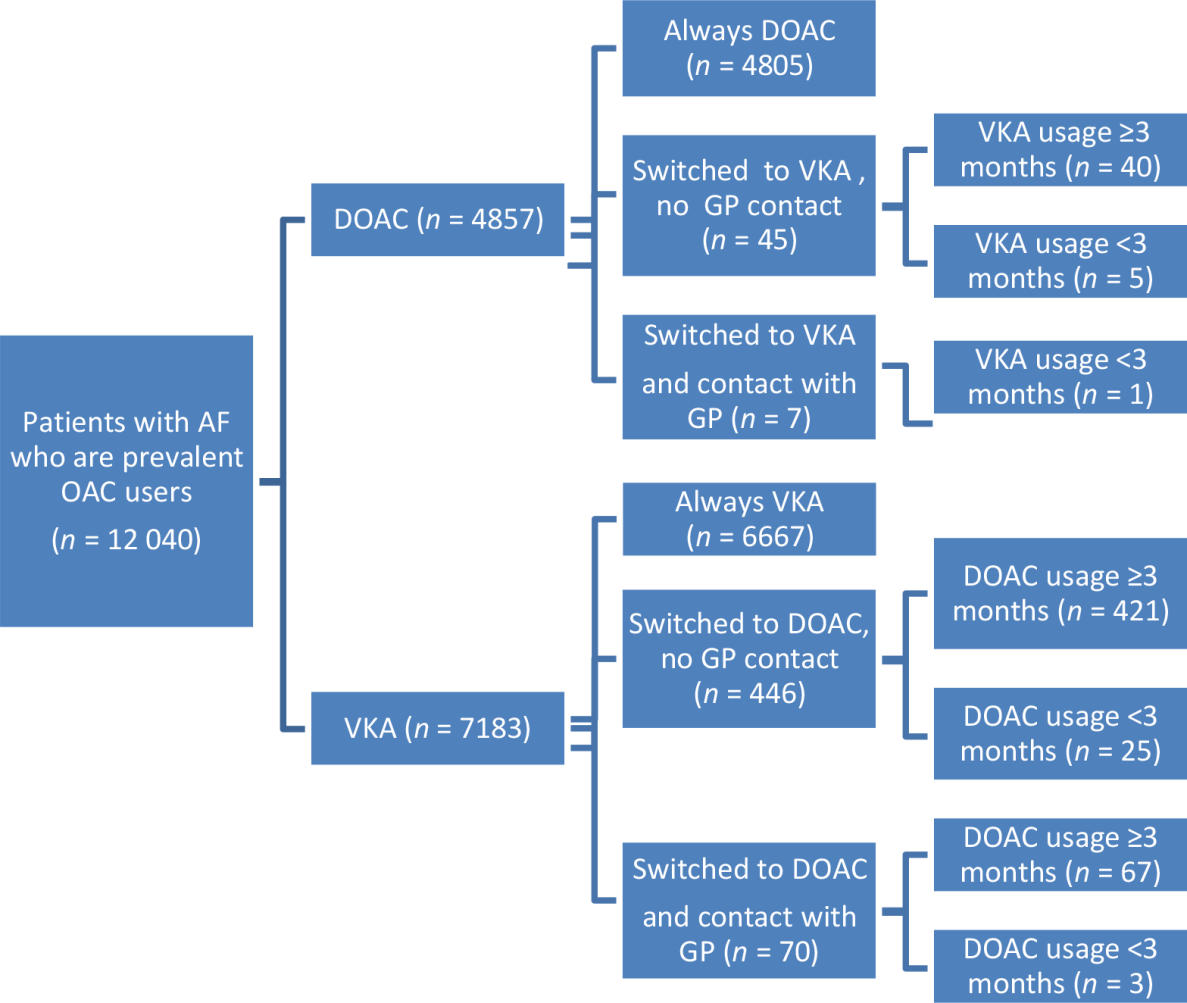
Prevalent patients with atrial fibrillation (AF) using oral anticoagulant (OAC) in 2018. DOAC = direct oral anticoagulant. VKA = vitamin K antagonist.

Of all prevalent users of OAC; almost 5% (*n* = 568/12 040) switched from one group to another, mostly from VKAs to DOACs (91%, 516 patients) (Figure 2). In 77 of the 568 patients who switched type of OAC, this switch was likely initiated by a GP, again mostly from VKAs to DOACs (0.9%; *n* = 70/7183). This was 0.6% (*n* = 77/12 040) of all prevalent users. After switching, new medication (that is, DOAC) was used at least months.

Looking at the prevalent VKA users, those who continued using VKAs had a higher percentage of heart failure comorbidity compared with prevalent VKA users who switched to DOACs.

## Discussion

### Summary

This study has demonstrated that, when starting an OAC for patients with AF. Dutch GPs mainly prescribe DOACs. The majority of prevalent OAC users used VKAs. For both starters and prevalent users, switching between type of OAC was not common. However, those patients who did switch, remained on the new type of OAC for at least 3 months.

### Strengths and limitations

This study had several strengths. A large representative routine care database for the Dutch population was used with a good reflection of prescribing behaviour of GPs. Since a longitudinal database was used, switching could be assessed during multiple years. A few limitations are also acknowledged. First, to identify prescriptions initiated by a GP, prescriptions were only included when there was a contact with a GP on the same day or previous day of the prescription date, since the study was concerned with GP prescribing patterns. A large number of patients were excluded (1552 patients). Therefore, the number of prescriptions initiated in primary care might have been over- or under-estimated. However, the sensitivity analysis in a subset of practices in which the prescriber was known confirmed the findings. Second, in the data not many patients switched their OAC treatment. Prevalent users might have already switched their OAC before the study period. Therefore, patient characteristics related to switching of medication could not be identified. However, other research also showed a low rate of switching to a DOAC for patients treated with the VKA warfarin, who were eligible to be switched to a DOAC.^24^

### Comparison with existing literature

It has been shown that switching rate overall between OAC in general practice is not common. An Australian cohort study showed a switching rate of 5.7%,^25^ matching the present study's switching rate of 5% of prevalent users and 6% switching rate among starters. A Canadian study found a switching rate of 7.8% with most of them being from warfarin to DOAC.^26^ However, an American study found much higher switching rates. But just like the present study, overall, switching to a DOAC occurred more frequently than switching to a VKA.^27^

French research on switching OAC in patients with AF showed treatment satisfaction was not only greater when starting with a DOAC compared with a VKA, but also when switching to a DOAC after first using a VKA. As in the present study, very few people in that study switched from a DOAC to a VKA.^28^ Also Toorop *et al* ^29^ showed that, in a group of patients of whom the majority received a DOAC because of AF, DOAC treatment satisfaction was high for patients who previously used a VKA.

Dutch GPs show a preference for prescribing DOACs in patients newly diagnosed with AF, although in the guideline a specific type of OAC is not advised. This was also found in the UK. Before the National Institute for Health and Care Excellence (NICE) guideline on AF was published in 2021 advising DOACs, a preference for DOACs was already found. In the UK, prescribing of DOACs in primary care increased from 9% of all anticoagulants in 2014 to 74% in 2019, while prescribing of warfarin declined accordingly.^30^

### Implications for research and practice

The data showed that in Dutch primary care most patients with AF on starting OAC are prescribed DOACs and continue using DOACs. In time, this will result in an increasing number of DOAC users and a decreasing number of VKA users. In the long term, this might endanger the sustainability of thrombosis services, as is the current standard for frequent VKA monitoring in The Netherlands. Frequent monitoring will always be needed for those patients using VKAs; that is, those with a contraindication for DOACs. This research could not determine what proportion of the patients had contraindications for DOACs. However, in future management, it is important to know more about this group. Patients who use DOACs will also need regular monitoring, although less frequently than VKA users. It is advised to evaluate renal function before initiation of a DOAC and to re-evaluate when clinically indicated and at least annually. However, these most necessary controls for DOAC users are not yet centrally organised in the Netherlands. This monitoring can be undertaken by GPs, in secondary care or in specialised AF clinics.

Further exploration is needed on whether VKA and DOAC monitoring can be combined in the future to reflect the way VKA monitoring is currently organised.

In conclusion, it was found that the majority of patients with AF, when starting OAC in primary care, start with DOACs, and that switching OAC by their GPs was uncommon in the study period. Centrally organised follow-up of patients using DOACs is not yet organised. These points stress the need for the organisation of check-ups for patients using DOACs. How to organise this and if this could be combined with VKA check-ups needs further research.

## Data

The dataset relied on in this article is available from the corresponding author on reasonable request.
